# Fees and Secret Commissions in Medical Practice

**Published:** 1906-03-10

**Authors:** 


### Fees and Secret Commissions in Medical Practice.

The public washing of dirty linen is proverbially an inelegant and unedifying process, and those who undertake the task must expect to have their motives impugned and questioned. Nevertheless such a proceeding may be necessary, and indeed praiseworthy, both in the interests of the body politic and also to avoid direct or indirect responsibility for the concealment of moral or legal crime. When, therefore, some man of strong and unblemished character whose record and aims are beyond suspicion, lays bare to the public gaze a scandal which touches the good name of the profession to which he belongs, men who love honesty more than selfish interest will be inclined to lend an attentive ear. Such, it seems to us, must be the attitude that must be adopted by the medical profession towards some statements recently made by Dr. T. H. Halstead when vacating the presidential chair of the Syracuse Academy of Medicine. Briefly the sum of Dr. Halstead's charge is that there exists in the United States, though by no means universally, a practice which is not only derogatory to the honour of the medical profession, but is at variance with the claims of common honesty. This is the custom on the part of certain consultants of paying to the family medical attendant on whose advice their opinion has been requested a proportion of the fee which they receive, the transaction being secret and entirely unknown to the patient. Obviously the gravamen of the charge lies in the last clause. Were such a method of arranging fees an open and declared custom, there is nothing more to be said. But for a consultant to receive as *his* fee a sum which he intends secretly to divide with the family adviser is deliberately to mislead the patient and to introduce into professional relationships the dangers and taint of a practice which, even in the ruder world of commerce, is condemned as immoral and dishonest.

Public confidence in the medical profession rests on the belief that medical advice is determined by a consideration of the welfare of the patient, and not by the financial interests of the doctor. When this ceases to be true, medicine ceases to be a profession, and becomes a selfish and sordid occupation, and one utterly repugnant to refined and self-respecting minds. It is because Dr. Halstead sees plainly, not only the dishonesty of the practice which he has denounced, but also its tendency to weaken the entire moral fibre of those who practise it that he has felt it his duty boldly to speak out, and in so doing he has, in our judgment, deserved well of all who recognise the true responsibility and essential dignity of honest medical practice.

In this country it can hardly be necessary to argue in support of the position which Dr. Halstead has adopted. Possibly some may urge that he should have proceeded rather by private remonstrance than by public condemnation. But with this we cannot agree. The practice in question can only arise from so complete and vicious a misconception of the whole attitude of the profession towards patients and fees that anything short of open denunciation could not hope to be effective. It is a case for radical and severe treatment, and this, we are glad to see, it has received. Further, we have no doubt that the general voice of the profession, on the other side of the Atlantic as well as on this, will endorse Dr. Halstead's method as well as his position, and will condemn in the most severe manner a practice which is nothing more or less than juggling with fees.

More than any other conviction the profession generally holds that it is necessary to keep medical practice free from the risks of commercialism, and, in spite of the aberrations of a few individuals, there can be no doubt that any departure from this attitude would be resolutely opposed. Quite recently we heard of a proposal from one misguided member of a hospital staff to his colleagues to found a Zollverein or consultations union for their mutual benefit, and it is needless to describe the manner of his reception. Our readers will readily recall the outspoken condemnation of unnecessary operations by a distinguished specialist some few years ago and the chorus of approval which his words commanded. The medical profession will always support efforts to keep its relationships to the public and to individual patients pure and true and of good report, and because Dr. Halstead's strictures have this aim and motive we congratulate him on the courage and effectiveness with which he has accomplished a difficult and ungrateful task.

